# Inorganic Polymer Matrix Composite Strength Related to Interface Condition

**DOI:** 10.3390/ma2042216

**Published:** 2009-12-07

**Authors:** Donald W. Radford, Andrew Grabher, John Bridge

**Affiliations:** 1 Composite Materials, Manufacture and Structures Laboratory, Mechanical Engineering, Colorado State Universtiy, Fort Collins, 80523-1374 Colorado, USA; E-Mail: eddy_win13@hotmail.com (A.G.); 2 Maine Maritime Academy, Castine, 04420 Maine, USA; E-Mail: john.bridge@mma.edu (J.B.)

**Keywords:** geopolymer, inorganic polymer, ceramic matrix composite, engine valve

## Abstract

Resin transfer molding of an inorganic polymer binder was successfully demonstrated in the preparation of ceramic fiber reinforced engine exhaust valves. Unfortunately, in the preliminary processing trials, the resulting composite valves were too brittle for in-engine evaluation. To address this limited toughness, the effectiveness of a modified fiber-matrix interface is investigated through the use of carbon as a model material fiber coating. After sequential heat treatments composites molded from uncoated and carbon-coated fibers are compared using room temperature 3-point bend testing. Carbon-coated Nextel fiber reinforced geopolymer composites demonstrated a 50% improvement in strength, *versus* that of the uncoated fiber reinforced composites, after the 250 °C postcure.

## 1. Introduction

The development of fiber reinforced composite materials technology for elevated temperature applications is most often limited by toughness and manufacturability. High temperature polyimide matrix composites have use temperatures approaching 400 °C. These polymers have been tailored for resin transfer molding (RTM) and resin infusion molding. Fiber reinforced polyimide engine intake valves have been under investigation for a number of years, and successful prototypes have been produced using RTM [1,2,3]. While the potential exists to survive the temperatures of the intake valve using these high temperature polymers, the roughly 800 °C requirement for an exhaust valve is clearly out of reach for these polyimides.

**Figure 1:**
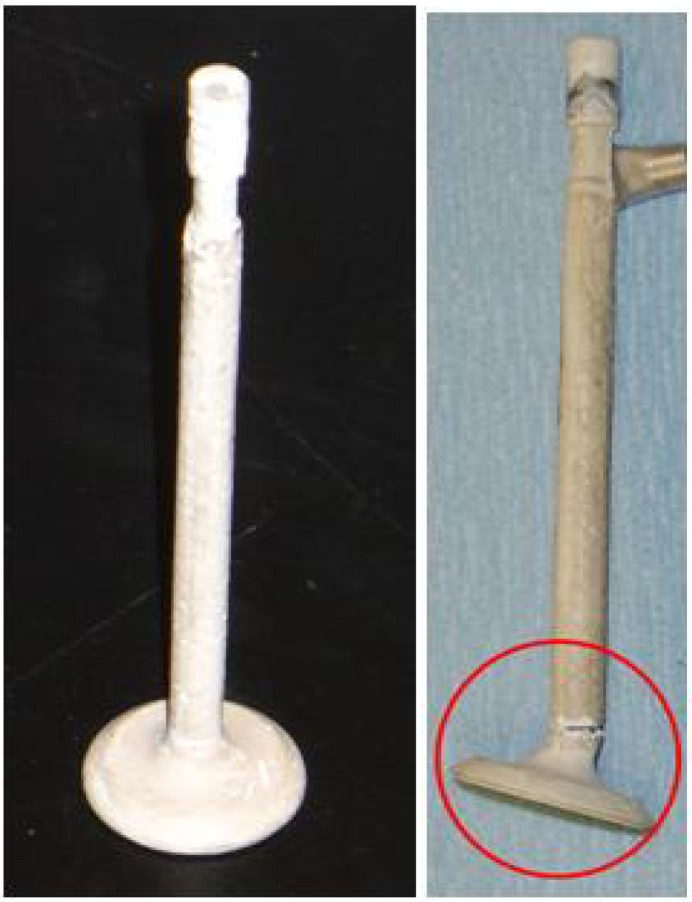
Resin Transfer Molded, Nextel 440/MEYEB composite Engine Valve and brittle handling failure.

Based on successful RTM development of intake valve prototypes, liquid geopolymer resin was considered as a candidate material for exhaust valve application. The commercially available MEYEB FS resin and MEYEB hardener were obtained from CORDI-Géopolymère^®^, and attempts were undertaken to mold ceramic fiber reinforced/MEYEB™ matrix composite valves using a RTM approach. The resulting molded valves, as shown in Figure 1, demonstrated the potential for RTM as a processing technique for this liquid inorganic polymer resin. The molded composite valves were well wet out and had good shape retention. However, the components were too brittle to be considered for in-engine testing, and in the case shown in Figure 1, failed during removal from the RTM mold. Thus, a laboratory mechanical test project was undertaken to investigate the potential for enhancement of the toughness of these ceramic fiber reinforced inorganic polymer matrix composites.

## 2. Background

“Geopolymer” is the term often used to refer to a class of alumina-silica based inorganic materials which are processed like a polymer that undergoes polycondensation at low temperatures, but resembles ceramics in the resulting structure and high temperature resistant properties [4,5]. The minerals that constitute this material are readily available. The name “geopolymer” was coined in 1978 during research efforts focused on the development of fire-resistant, non-toxic materials to be used in building structures [6]. This material evolved into a mineral-based binder for use as a high strength industrial cement with significantly shorter cure times than traditional Portland cements [4]. More recently there has been interest in utilizing the high-temperature resistant properties of this material and its very low density to replace heavier metallic components in high-temperature applications. The ease of processing fiber reinforced composites with these inorganic polymer binders as the matrix, compared to traditional ceramic matrix composites (CMC’s), also makes them attractive. The samples used in this study were prepared using standard composite fabric wet-layup techniques, and initially cured at 80 °C for 1 hour followed by a freestanding postcure at 250 °C for 5 hours. The result is a matrix material with stable mechanical properties to temperatures in excess of 750 °C. This is in contrast to traditional ceramic matrix composite manufacturing processes which generally require much higher processing temperatures, usually exceeding 1,000 °C [7]. While a substantial amount of research has been published on cementatious variants of these inorganic polymers, there is limited data available on composites produced using the thermoset resin-like versions, such as the MEYEB FS resin used in resin transfer molding of ceramic fiber reinforced engine valves.

### 2.1. Chemical Structure of the Inorganic Resin

The geopolymer can exhibit several different structures characterized by tetrahedral aluminate and silicate units referred to as “aluminosilicates” [4]. The aluminosilicate starting precursors, the AlO_4_ and SiO_4_ tetrahedra, are found naturally in the mineral metakaolinite (nominal composition Al_2_O_3_·2SiO_2_,) by calcining kaolinite at 700 °C to remove chemically attached water [8]. The precursors are polycondensed with alkali activators, either KOH or NaOH, to form structures which may be amorphous or semicrystalline. These resulting structures are charge-balanced by the addition of alkali metal ions such as sodium and potassium [5]. Three prominent structural units exist, with the base unit referred to as “sialate”, which is an abbreviation for silicon-oxo-aluminate [4,9]:

(1) “sialate” [-Si-O-Al-O-]

(2) “sialate-siloxo” [-Si-O-Al-O-Si-O-]

(3) “sialate-disiloxo” [-Si-O-Al-O-Si-O-Si-O-]

These sialate-based fragments condense together to form larger polymeric structures called polysialate (PS), polysialate-siloxo (PSS), and polysialate-disiloxo (PSDS) [10,11]. The sialate network structure containing the SiO_4_ and AlO_4_ tetrahedra units are linked in an alternate fashion by the sharing of oxygen atoms. To balance the negative charge of Al^3+^ in IV-fold coordination, positive ions (Na^+^, K^+^, Li^+^, Ca^++^, Ba^++^, NH^4+^, H_3_O^+^) must be present in the structural spaces [4,5]. The structure of the MEYEB inorganic polymer resin used for this study is PS containing potassium ions and is predominantly amorphous.

Like many organic polymers, this inorganic polymer polycondenses in just a matter of minutes. The mechanical properties of the matrix appear to be directly related to the silica and alumina ratio - higher silica leads to higher strength [4,12].

### 2.2. Ceramic Fiber Reinforced Inorganic Polymer Matrix Composites

The incorporation of ceramic fibers can significantly strengthen the geopolymer. In one study, adding 11.3% by volume, 3 mm-long short alumina fibers to a geopolymer matrix resulted in an almost 10-fold improvement over the addition of 9 vol.% alumina fiber paper to the same matrix material (97 MPa *vs.* 16 MPa) [13]. Using SiC plain weave fabric with a potassium-based PSS geopolymer, a maximum flexural strength of 380 MPa is reported [4]. At temperatures up to 1,000 °C, SiC fabric-PSDS geopolymer strengths are reported to range between approximately 225 MPa at 100 °C and 110 MPa at 1,000 °C [4].

## 3. Experimental

The current research focuses on the measurement of the effects of fiber-matrix interface modification on the strength of Nextel 440 reinforced MEYEB inorganic polymer matrix composites. The Nextel 440 ceramic fiber is a combination of alumina and mullite, and the MEYEB inorganic resin cures to an aluminosilicate. The similarity of this fiber and matrix is expected to result in a very strong interface, and a correspondingly low toughness, especially after exposure to elevated temperatures, which can lead to even greater fiber-matrix interface strength. The choice of this fiber was made to evaluate the potential for interface modification in what might be considered a “worst case” combination of fiber and matrix.

The as-received Nextel 440 fabric has a polymer sizing on the fibers to protect the material from abrasion damage prior to incorporation into a matrix. To investigate the effectiveness of interfacial strength modification, as-received Nextel 440 fabric was treated in two different ways. In both cases the fabric was heated to 700 °C. In one case the atmosphere was air, resulting in a cleaned fiber surface, while in the other case the atmosphere was N_2_, resulting in a carbon coating. The fabric with the carbon coating on the fibers is predicted to result in a composite with reduced interfacial bond strength, and therefore, increased strength, as long as the carbon layer remains intact. Laminates were produced, in an otherwise identical fashion, using the two reinforcement modifications and the MEYEB inorganic polymer as the matrix. Bend beams were created and heat treated to different temperatures, before testing at room temperature, to investigate the effectiveness of fiber-matrix interface modification and the response of the interface, in both cases, to time at temperature.

### 3.1. Materials

Two laminates, each nominally 108 mm × 108 mm, were produced from Nextel 440 fabric and the MEYEB inorganic polymer by wet layup. One laminate was composed of fabric that had been cleaned and the other incorporated fabric with the carbon coating. Eight plies of the Nextel 440, 5-harness satin weave fabric with 2,000 denier roving, resulted in a nominal cured thickness of 2.8 mm.

**Figure 2:**
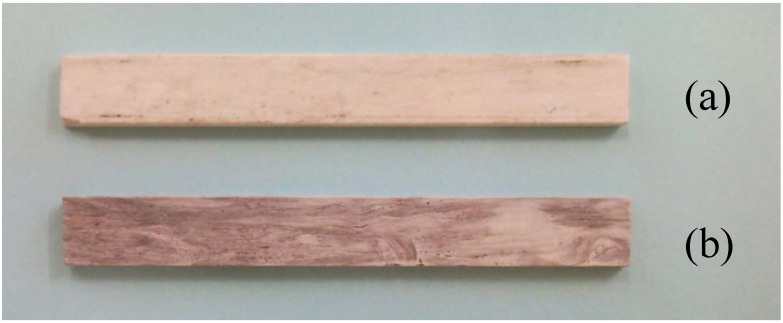
Representative samples of the specimens prepared for bend testing; (a) fibers cleaned, and (b) fibers heat treated in Nitrogen to develop a carbon coating.

The MEYEB resin must be stored at a temperature of −18 °C or below, but once mixed it has a room temperature use life of about 30 minutes. Thus, the laminate size and number of plies was controlled to enable completion of lamination within the 30 minute time period. The MEYEB inorganic polymer was mixed with the supplied hardener at a ratio of 6.7:1 by weight. The individual fabric layers were wet-out with a pre-measured amount of mixed MEYEB resin, targeting a fiber volume fraction (Vf) of 50%. The plies were stacked, one on top of another, to create two 4-ply (0/90) laminates. One of the 4-ply laminates was then inverted and placed on top of the other 4-ply laminate to create a symmetric 8-ply (0/90) laminate. Inverting the 4-ply sub-laminate of the woven fabric was done to ensure that fabric nesting did not result in laminate asymmetry, and associated distortion at temperature. The laminate was then transferred to a hot press and heated to 80 °C at approximately 5 °C/min, under a pressure of 690 kPa. The laminate was cured for 1 hour at 80 °C, followed by a 5 hour postcure at 250 °C in the hot press. An important by-product of hot press processing was the generation of two smooth, parallel surfaces which would become the top and bottom of the 3-point bend test specimens. The resulting postcured laminates were measured to have a fiber volume fraction of 47%. The two laminates were visibly different in appearance, with the composite prepared from the cleaned fabric being uniformly white in color, while the specimen composed of the fabric treated in a Nitrogen environment had a definite grey tint, as seen in two samples shown in figure 2. This grey tint was also apparent on the fabric after heat treatment in the Nitrogen environment and is consistent with the generation of the carbon coating on the fibers.

### 3.2. Specimen Preparation

**Figure 3:**
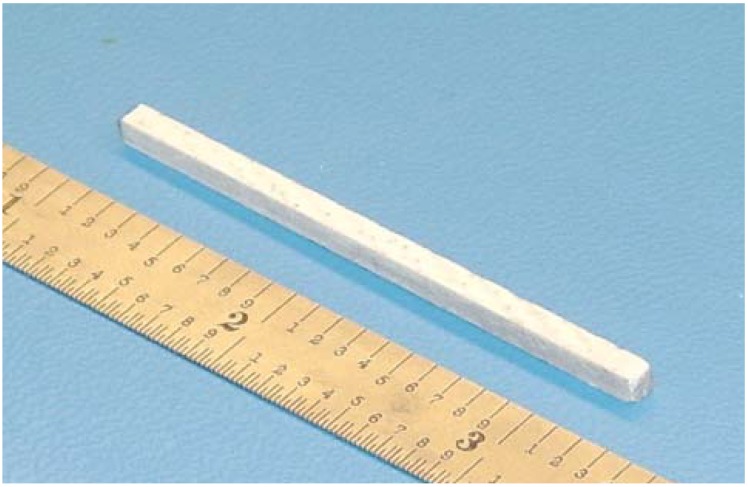
Finished bend beam specimen, nominally 56.5 mm L × 2.8 mm W × 2.8 mm T.

Bend beam specimens were prepared from each of the two laminates. Laminates were cut to a length of 56.5 mm and then this sub-laminate was cut into specimens of just over the desired 2.8 mm in width, using a wet diamond saw. The remainder of the 108 mm laminate was prepared for other short beam shear testing [14]. The specimens were dry sanded in three stages, on the cut edges, using a special fixture designed to maintain a square cross-section and generate a specified width. Samples were sanded with 240, 500, and 1,000 grit sand paper. Finished specimens were approximately 2.8 mm × 2.8 mm × 56.5 mm (thickness, width, length) as shown in Figure 3. Sanded edges were cleaned with denatured alcohol and a paper towel. The samples were then measured and weighed.

The tests included exposure of samples to high temperatures for varying amounts of time to evaluate the effect of temperature on the mechanical properties. To accomplish this, a group of specimens from each laminate was retained with only the 5 hour, 250 °C postcure, and then three other groups of specimens were heat treated in a refractory oven, in air. The three additional heat treatments were cumulative. The result was four sets of specimens: (a) 5 hours at 250 °C, (b) 5 hours at 250 °C, plus 5 hours at 650 °C, (c) 5 hours at 250 °C, 5 hours at 650 °C, plus 1 hour at 900 °C, and finally, (d) 5 hours at 250 °C, 5 hours at 650 °C, plus 10 hours at 900 °C. A minimum of three specimens were prepared for each condition evaluated.

**Figure 4:**
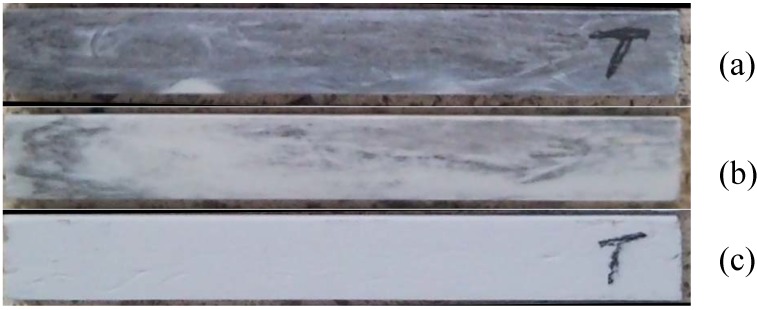
A single carbon coated Nextel 440 fiber reinforced MEYEB sample after exposure to: (a) 250 °C for 5 hrs, (b) 250 °C for 5 hrs + 650 °C for 5 hrs, and (c) 250 °C for 5 hrs + 650 °C for 5 hrs + 900 °C for 5 hrs, all in air.

It was expected that the carbon layer on the fibers of the Nextel 440 composite laminates would not be stable in air at elevated temperatures. Exposure to high temperatures may lead to deterioration of fibers, matrix, or the interfacial characteristics. Figure 4 shows three images of the same carbon-coated Nextel 440 reinforced sample after the preliminary postcure at 250 °C and then after each of the subsequent heat treatments. It is clear that a change took place as the grey color disappears with increasing temperature exposure, suggesting the loss of the carbon coating at the interface.

### 3.3. Bend Test Procedure

**Figure 5:**
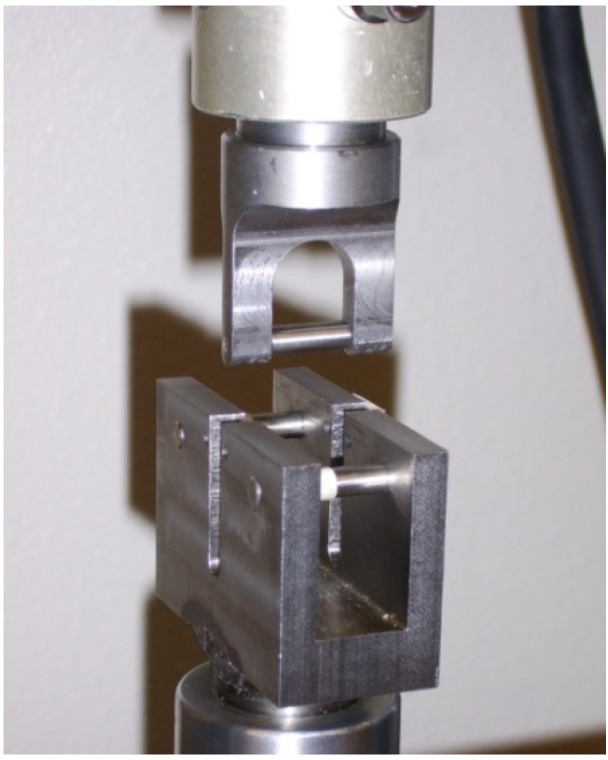
3-point flexural beam test fixture in loadframe. Test span is 45.4 mm.

The tests performed utilize a 3-point bend test fixture with a fixed span of approximately 45.4 mm. This results in a span to depth ratio of 16:1, which is considered sufficient to generate pure bending mode response for a woven fabric reinforced ceramic matrix composite [15]. Due to limitations related to the amount of material available, the specimens were narrower than specified in the ASTM standard [15]. The 3-point bend fixture shown in Figure 5 has support pins and a loading nose of 6.35 mm in diameter.

A crosshead rate of 1.25 mm/min was used for all tests, and the midspan deflection was monitored through calculation of crosshead travel, based on time and crosshead rate. The compliance of the load train was accounted for through measurements of both a steel and aluminum beam standard, using the approach outlined in the appendix of the ASTM standard. Stress at the bend specimen surface is calculated using the relationship:

(1) $$\sigma =\frac{3PL}{2bd^{2}}$$

where;

*σ* = maximum stress, at beam surface
*P* = measured load
*L* = span of the test fixture (45.4 mm)
*b* = beam width (nominally 2.8 mm)
*d* = beam depth (laminate thickness, nominally 2.8 mm)

A load cell with a maximum load range of 445 N was used for all tests and the load data were captured using computer data acquisition. The associated strain and modulus values are based on midspan displacements determined from the crosshead motion.

### 3.4. Scanning Electron Microscopy

Scanning electron microscopy of selected bend beam specimen fracture surfaces was undertaken to confirm the effect of the fiber surface modification. An Amray, AMR-1000 electron microscope was used. Specimen fracture surfaces were sputtered with carbon to create a thin coating to allow imaging.

## 4. Results and Discussion

### 4.1. Mechanical Properties

**Figure 6:**
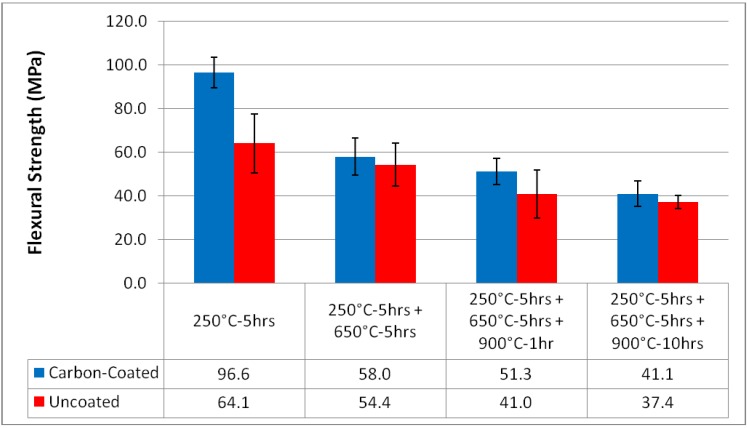
Mean Flexural Strength versus Heat Treatment and Interface Modification (error bars indicate ±1 standard deviation).

All specimen failures indicated tensile-mode failure at the outer surface of the specimen, and a relatively planar fracture surface. The resulting failure mean strengths and associated error bars equal to plus, and minus, one standard deviation, are shown in Figure 6. For both the carbon-coated fiber reinforced specimens and the uncoated fiber reinforced specimens the trend in strength is a reduction with increasing heat treatment temperature. The maximum average strength recorded, 96.6 MPa, was for the carbon-coated Nextel 440 reinforced MEYEB laminate, after the baseline postcure of 250 °C. The corresponding average strength of the cleaned fiber reinforced specimens after only the 250 °C postcure was 64.1 MPa.

For each thermal treatment the average strength of the composite reinforced with the cleaned fibers is lower than the corresponding value for the composite with the carbon-coated reinforcement. However, when considering the standard deviation for each set of results, there seems to be a significant difference in strength after 5 hours at 250 °C, but it is difficult to state absolutely that there is a difference in the strengths of the cleaned and carbon-coated fiber reinforced specimens after each higher temperature treatment. Clearly for both groups, cleaned and carbon-coated, the strength is reduced with thermal treatment. After the extended duration thermal treatment at 900 °C the strength of the specimens representing each fiber condition has fallen to approximately 40 MPa.

To test these observations, based on the mean and standard deviation of the measured specimen strength, a “two-sample t-test, assuming unequal variances” was applied. Based on a 95% confidence level, the strengths of the carbon-coated fiber reinforced specimens do decrease with temperature, when compared with the 250 °C for 5 hour conditioning. Further, while a drop in strength between 650 °C and 900 °C for 1 hour cannot be discerned, statistically, the t-test does indicate a difference in strength between 650 °C and 900 °C for 10 hours. Within the 95% confidence limit, the strengths of the cleaned fiber reinforced composites cannot be shown to decrease with temperature; however, if the confidence limit is relaxed, only slightly, then a difference between the mean strength at 250 °C and 900 °C after both 1 and 10 hours emerges. Statistical comparison of the carbon-coated and the cleaned specimens, after each thermal treatment, indicates that a significant difference exists only after the thermal treatment at 250 °C.

The drop in strength with increasing thermal treatment temperature is consistent with an increasing fiber-matrix interfacial strength and a reduction in toughness [16]. The highest strength for the carbon-coated fiber reinforced MEYEB after only the 250 °C postcure suggests that the reduced interfacial bond strength related to the carbon interphase region is effective in controlling crack propagation. Yet, with further thermal treatment in air, the amount of carbon at the interface is depleted and the interfacial strength increases as the two constituent materials develop ever increasing bond strengths. This increasing interfacial bond strength results in a composite with less ability to accommodate damage and correspondingly with a lower failure strength, as seen with increasing heat treatment temperature in figure 6. After the most extreme thermal conditioning, all of the carbon at the interface is depleted and there has been sufficient time at temperature that both composites behave in a very similar manner, are expected to have very similar interfacial characteristics, and show statistically identical failure strengths. The drop in the strength of the uncoated fiber reinforced specimens is consistent with an increasing bond strength driven by interdiffusion of the fiber and matrix, due to the similar structures [16].

**Figure 7:**
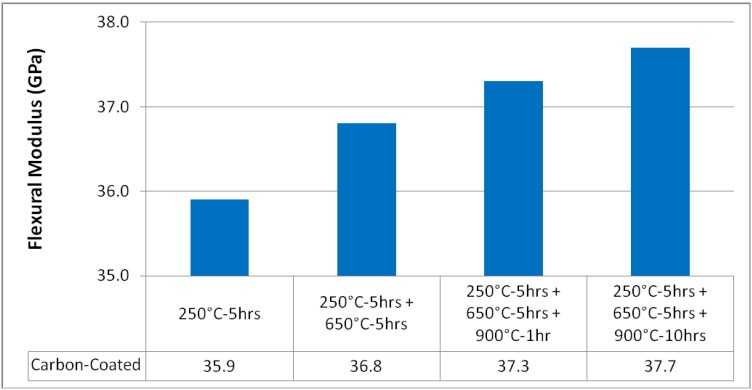
Mean Flexural Modulus versus Heat Treatment for Carbon Coated Fibers.

To further evaluate the effect of the changes in the carbon-coated fiber reinforced MEYEB, the measured modulus can be evaluated. As shown in Figure 7, the modulus for the composites based on the carbon-coated Nextel fabric increases from 35.9 GPa after the 250 °C postcure, to 37.7 GPa after the extended exposure at 900 °C. The standard deviation in the modulus measurements was less than 2% and thus is not shown in Figure 7. This measured increase in modulus, with an assumed increase in fiber-matrix interfacial strength, is consistent with improved load transfer at the interface.

### 4.2. Fracture Morphology

**Figure 8:**
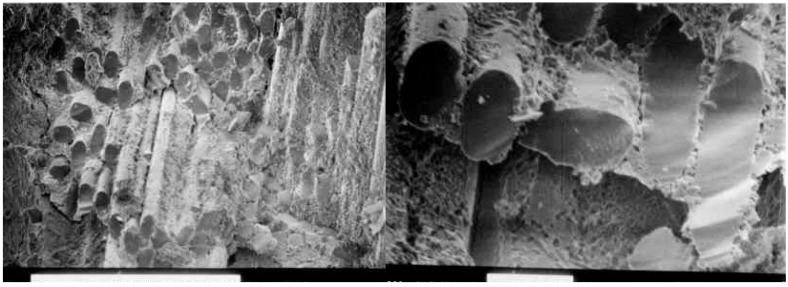
SEM Fracture Surface: Uncoated fiber surfaces tested after 250 °C postcure.

**Figure 9:**
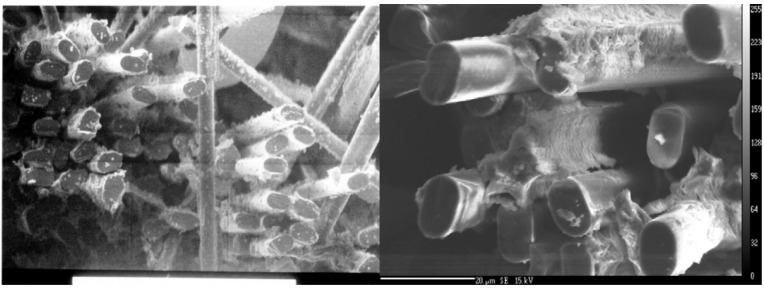
SEM Fracture Surface: Carbon-coated fiber surfaces tested after 250 °C postcure.

To further investigate the effectiveness of the carbon-coating at the fiber-matrix interface, scanning electron microscopy of the fracture surfaces was performed. Figure 8 and Figure 9 show the fracture surfaces of composites without the carbon-coating at the interface, and with the carbon-coating, at two different magnifications. It is clear in Figure 8 that the fracture surfaces are planar, traveling from the matrix through the fibers and back into the matrix. Conversely, Figure 9 clearly shows fiber pullout, including sockets in the matrix where the broken fiber has been cleanly extracted. This fiber pullout is consistent with a reduced interfacial strength and with an increased energy of fracture.

### 4.3. Future Tests

Future work is currently planned to process and test samples from the same geopolymer-based high temperature resin and various unidirectional reinforcements, including alumina (Nextel) and SiC fibers. The use of uni-directional reinforcement will simplify the structure of the composite, compared to the woven fabric specimens of the current work, and enable improved prediction of the interfacial strengths. These composites will be tested to evaluate various fiber/matrix interface conditions, including interfacial coatings which have elevated temperature stability in air. Unlike the current tests that were performed at room temperature, future testing will include elevated test temperatures. Both 3-point bend tests and dynamic mechanical analysis in a 3-point bend configuration are planned.

## 5. Conclusions

The limited toughness of the Nextel 440/MEYEB resin transfer molded engine valves resulted in the evaluation of the effect of interfacial strength modification and testing. The bend specimens treated with a carbon layer to reduce the interfacial strength did show an improvement in flexural strength, consistent with an increase in toughness. The 50% increase in strength achieved through this simple modification is promising, as it supports the notion that methods of toughness enhancement successful in more common ceramic matrix composites can be directly applied to composites using this inorganic polymer binder as a matrix material. It also suggests a path for the successful utilization of reinforcing fibers with similar chemical make-up to the matrix material, which in general is a worst case scenario for ceramic matrix composite toughness. Further, unlike many ceramic matrix composites which are processed at, or above the planned use temperature, the MEYEB matrix composites are processed at temperatures that may be substantially lower than the use temperature. This relatively low temperature processing is very beneficial in the creation of composite components, but results in an as-processed composite strength that may be negatively affected as the use temperature exceeds the postcure temperature. Thus, the results of the research support the ability to improve the toughness of such inorganic polymer matrix composites, with the goal of resin transfer moldable, high temperature structural composites.
